# Communication Styles and Attention Performance in Primary School Children

**DOI:** 10.3390/bs11120172

**Published:** 2021-12-09

**Authors:** Gabriel G. de la Torre, Miguel A. Ramallo, Sara Gonzalez-Torre, Alvaro Mora Prat, Andrea Rueda-Marroquin, Amanda Sallago-Marcos, Zoraida Toro-Barrios, Manuel A. Garcia

**Affiliations:** 1 Neuropsychology and Experimental Psychology Lab, Campus Rio San Pedro, University of Cadiz, Puerto Real, 11510 Cádiz, Spain; miguelangel.ramallo@gm.uca.es (M.A.R.); sara.gtm@uca.es (S.G.-T.); mantonio.garcia@uca.es (M.A.G.); 2 Biomedical Research and Innovation Institute of Cádiz (INiBICA), 11006 Cádiz, Spain; 3 University Hospital Doctor Peset, 46017 Valencia, Spain; alvaromora.psicologia@gmail.com; 4 Department of Psychology, University of Cadiz, 11510 Cádiz, Spain; andrearuema8@gmail.com; 5 European University of Valencia, 46010 Valencia, Spain; amandasallagomarcos@gmail.com; 6 University of Huelva, 21007 Huelva, Spain; zoraidatoro@outlook.es; 7 University Research Institute for Sustainable Social Development (INDESS), Avda. Universidad, 4. Campus de Jerez, Jerez de la Frontera, 11405 Cádiz, Spain

**Keywords:** communication styles, attention, primary school, assertiveness

## Abstract

Communication styles are the three communication patterns that result from merging the verbal elements of communication, the non-verbal elements and the paraverbal elements. The objective of this study was to test what effect different communication styles have on attention performance in primary school children. We administered the assertive behavior scale for children (CABS), the symbol digit modalities test (SDMT) and the d2 attention test to a sample of 77 participants of primary school. A statistically significant and proportional correlation was found between the assertive communication style and the total number of correct answers of the SDMT. We also found positive correlations between the assertive communication style and d2 attention test performance. Children with an assertive communication style have better attention performance than children with passive or aggressive communication styles.

## 1. Introduction

Communication styles are behaviors that consistently occur in “the way one verbally, nonverbally, and paraverbally interacts to signal others how literal meaning should be taken, interpreted, filtered, or understood” [1]. Each of us has our own styles of communication; some facilitate relationships with others, while others make it difficult to communicate and develop a good personal relationship with others. Communication styles influence the way people interact, exchange information, support, collaborate, learn collectively, etc. [2,3,4,5].

During the development stages, and especially during puberty, some skills are acquired, such as social skills, emotional control and self-control of behavior that allow us to participate in everyday activities. Among these skills, we can also include the empathy and communication styles [6]. Studies analyzing the relationship between empathy and acceptance in peer groups show that children with a high level of acceptance by their peers are more empathic. Accepted children show a more positive orientation to others and a strong sensitivity for the anguish of others [7], and prosocial children have a greater empathic knowledge than stalkers or those who are victims of the latter [8]. Another line of research has shown that empathic children and adolescents have greater emotional stability. During childhood, the emotional components of happiness and anger are correlated with empathy [9]. Some studies on adolescents suggest that emotional stability is a fundamental factor in establishing relationships of empathy [10].

We can identify three types of interpersonal communication styles: aggressive, passive and assertive [11]. A person with an aggressive communication style is characterized by defending their rights at any price; usually, this means not respecting the rights of others, even if it is unnecessary. People classified as aggressive do not take into consideration the rights of others. They also aim to reach their goals immediately without considering the negative consequences in the long term. That is, an aggressive interaction and communication style portrays individuals who base everything on their own rights, achieving their goals at the expense of others. Similarly, since the 1980s, different studies have argued and demonstrated that aggressive children have a deficit in perceiving and decoding environmental stimuli in an “unbiased”, realistic way [12,13,14,15,16,17]. Aggressive behavior is associated with biases in different aspects of attention [18], and this makes people with this style present problems to mitigate their hostile interpretations because they do not pay enough attention to environmental clues and may not encode the signals properly [19].

Recent studies warn that communication styles influence school performance [20,21]. This could be related to deficits in recognizing the stimuli of the environment. Additionally, several studies on impulsivity indicate a relationship between aggressive communication style and higher probability of showing more aggressive responses and behaviors [22,23]. The behavioral repertoires of these children consist of a smaller number of alternatives, added to the fact that they do not anticipate the consequences, which often leads them to choose the one that requires the least effort at that given moment. This response tendency, therefore, will offer a greater percentage of aggressive responses incompetent to “provocative” social situations and subsequently, the number of competent assertive responses would decrease. Thus, and in agreement with Deluty [24] and Spivack and Shure [25], children with aggressive styles are capable of conceiving assertive solutions, but the percentage of assertive alternatives in their repertoire is scarce. The greater the percentage of aggressive responses, the more aggressive the alternative according to the percentage [26].

The passive communication style involves not defending one’s own rights, not being able to express feelings, thoughts and opinions, and showing conformity to everything. The person with this communicative style lives preoccupied with satisfying others and avoids confrontation. In addition, he/she is a prudent and educated person. This type of person may generate feelings of helplessness, depression and tension in maintaining interpersonal and social relationships [11]

Assertive behavior consists of knowing what one’s personal rights and interests are and knowing how to defend them by using a series of skills that allow a subject to be objective and respectful of oneself and others without resorting to manipulation or pretense. The assertive person is able to express his/her feelings, ideas and opinions, as well as helping others to express themselves freely. The goal of assertive behavior is not only to achieve what is desired, but also to develop the negotiation skills necessary to achieve it. It is common in people with self-esteem and self-confidence [27,28,29,30,31,32,33,34]. Authors such as García Pérez and Magaz Lago [35,36], differentiated two characteristics of all assertive behavior: self-assertiveness, or the degree to which a person grants himself or herself the basic assertive rights or the degree to which a person considers that others have these rights, understanding basic assertive rights as those beliefs that facilitate the assertive behavior of people.

Attention is a cognitive process that can facilitate interaction with our environment and allows us receive information from all around us. For García [37] attention is a mechanism that sets in motion a series of processes or operations, thanks to which we are more receptive to the events of the environment and carry out a large number of tasks more effectively.

Wheeler and Carlson [38] suggested that attention deficit hyperactivity disorder (ADHD) children with a predominance of inattention profile may have limited social interaction and this aspect would restrict the acquisition of adequate social skills knowledge. They used the assertive behavior scale for children (CABS) to obtain scores from parents and teachers on the social status and social performance of children with ADHD and at the same time, they also used it to obtain scores on social knowledge and performance from children. They found differences between the different subgroups of ADHD children; on the one hand, the children with combined ADHD showed more aggressive responses, while on the other hand, it was perceived that the ADHD children with a predominance of inattention symptoms showed greater social passivity [39] ADHD children show a significantly more cognitive style of field dependence, which indicates how inattention would be associated with a lower level of assertiveness [40].

In the work carried out by Meza [41], it was revealed how assertiveness correlated with cognitive styles, as women who had greater field independence were more assertive. Guisande, Páramo, Tinajero and Almeida [42] showed how people with a greater degree of field independence focus their attention more analytically and have better control over the distractions that produce irrelevant stimuli than those dependent on the field.

According to this cognitive–behavioral conceptualization, which focuses on child interaction styles in relation to information processing, efforts were dedicated to evaluating the present solutions in the behavioral repertoire of children in order to establish their level of adaptation and competence. From this perspective, the first infant self-report tests on assertive behavior were modified versions of adult inventories, such as the Rathus assertiveness scale [43], with adaptations for high school students [44] and primary school students [45].

Reardon, Hersen, Bellack and Foley [46] created the self-report assertiveness test for boys (SRAT-B), with the aim of evaluating the answers that could be used in real life. Deluty [24] reported on the characteristics of the children’s active tendency scale (CATS), an auto report on aggressiveness, assertiveness and submission whose objective was to assess interpersonal conflicts in specific situations. However, this scale, like the previous one, showed a very low level of external validity.

Michelson and Wood [47] developed the assertive behavior scale for children (CABS), which aimed to classify aggressive, inhibited or passive, and assertive children. The original scale consists of 27 items, each with five response alternatives: aggressive, assertive, highly inhibited, inhibited and assertive, showing acceptable levels of consistency. The CABS also offers the possibility of contrasting individuals’ self-rating of relational styles with heterorating by significant adults and peers, in coherence with investigations that advocate the measurement of social competence from diverse sources [48,49,50,51]. They proposed in this scale a series of situations on topics such as making and receiving compliments, presenting and accepting criticisms, asking and giving things, accepting guilts, providing help, initiating conversations and behavior before orders. Michelson and Wood [47] found that CABS had good discriminant validity, as it was able to accurately differentiate in a group of 80 fourth grade children among those who had received social skills training from those who had not. Furthermore, the teachers, who were unaware of the treatment conditions, rated the children who had been in the social skills training group significantly more competent than those who did not. Not only that, but they were also able to identify the children who received 16 h compared to those who only received 8 h of social skills training. Thus, CABS was not only able to discriminate trained children from untrained children, but was also able to distinguish children who received 8 h of training from another group with 16 h of training.

The assertive behavior scale for children (CABS) was used to evaluate the effects that certain intervention programs had on the communication styles of different groups of participants ([47,52]). It was also used to perform research on the social impact of assertive versus non-assertive behavior in fourth and fifth grade children [53], as well as to compare communication styles in children with ADHD [39]. Johnson, Myers, Webber, Greenlund and Berenson [54] used the CABS to study assertive, aggressive and passive behaviors in relation to weight and other health-related risk factors for cardiovascular physiological and behavioral diseases. The CABS scale is a widely used scale to evaluate aggressive, passive and assertive communication styles in different areas and represents a helpful tool to assess the relationships between communication styles and school performance [20].

In line with these previous research studies using CABS and studying communication styles in children, the main objective of our study was to check whether assertive behavior increases the level of attention performance compared to the other communication styles in a sample of 77 children between 7 and 12 years old. This age period represents a critical period since executive functions and other functional processes develop and this was proved relevant in previous studies [9].

## 2. Method

To carry out the experiment, we initially had a sample of 83 children, but since 6 of them had to be considered null, the final sample consisted of a total of 77 participants. Of these 77, 48.1% were boys and 51.9% were girls. The mean age was 9.5 years (SD: 1.6).

The sample was collected in public schools of the southern region of Spain. In order to be included in the study, the children had to have Spanish as their first language, not present neurological or psychiatric damage or learning difficulties, and not be enrolled in the Special Education group. The participation of the students in the administration of the test was done voluntarily and with parents’ consents. From the initial sample, 6 participants were discarded because they did not understand the dynamics and instructions of the tests.

In order to access the sample, the management of the center was contacted, who informed the parents of the students, who authorized their evaluation. The students were evaluated within the class. There were always two evaluators who explained how to complete the questionnaires and were present throughout the process to be able to resolve the doubts that the children might have. To measure the different communication styles, the Spanish adaptation of the Wood and Michelson’s assertive behavior scale (CABS) [55] was used. Specifically, the CABS Type II was applied, consisting of 24 items adapted for schoolchildren in the upper elementary cycles. It is adapted with three possibilities of choice: assertive, aggressive and inhibited, instead of the five of the original scale as it was done in the Spanish adaptation [55]. The objective pursued with this adaptation was to achieve a short scale to be able to discriminate between the different communication styles; for this, the redundant items were eliminated and later, the item–test correlation of the remaining items that made up the scale was evaluated.

In order to evaluate aspects related to attention, we used two tests: the symbol digit modalities Test (SDMT) and the d2 attention test.

The symbol digit modalities test (SDMT) [56] consists of converting symbols in the shape of geometric figures into numbers, according to an established key. It consists of two forms: a written and an oral one. We used the written form for this study. According to Silva [57], the SDMT is one of the most recognized tests to evaluate information processing speed, and its multimodal essence with some executive and learning components and fast administration make it also a highly valuable tool for the detection of cognitive impairment.

The attention test d2 [58] is a time-limited test to measure selective attention and mental concentration, understood as the ability to selectively attend to certain relevant aspects of a task while ignoring the irrelevant (e.g., performing a selective search) and, in addition, to do it quickly and accurately. This test recently revealed positive associations between physical fitness, cognitive functioning and academic performance in children [59].

First of all, to carry out our study, permission was requested from the school management to conduct this experiment, and informed consent from parents was obtained. The experiment was carried out in accordance with The Code of Ethics of the World Medical Association (Declaration of Helsinki) for experiments involving humans.

In Spain, the educational system is divided into several levels: infant education, which is voluntary and is between 0 years and 6 years old, and the 0–3-year cycle is voluntary. Primary education comprises six grades (first to sixth) and includes children from 6 years to 12 years old. Compulsory secondary education (ESO), which is the last compulsory stage, includes children from 12 years to 16 years old. In the present study, the tests were administered to a group of children between 7 years and 9 years old (3rd grade) and another group of children between 10 years and 12 years old (6th grade of primary school). The first test administered was the d2. In this test, the participants crossed out the letters “d” with two selected stripes. There were 14 lines, and for each line the subject had a limited time of 20 s to find the maximum target stimulus.

After completing the d2, participants completed the assertive behavior scale (CABS II). This test consists of 24 items composed of several alternative responses: assertive, aggressive and inhibited. In this case, there was no need for a limited time. Finally, the symbol digit modalities test (SDMT) was administered. The SDMT consists of identifying the number associated with a given symbol over a period of time (90 s). Between tests, a rest period of approximately five minutes was offered.

The statistical analysis was carried out with the SPPSS version 22. First, descriptive analysis of the sample was carried out (Table 1). Next, we evaluated if the sample had a normal distribution. For this, the Kolmogorov–Smirnov and Shapiro–Wilk test was used, where it was observed that the distribution was not normal; due to this, for the subsequent analysis, non-parametric tests were used. Anonymized data were used for the analysis.

## 3. Results

In the first place, we carried out a descriptive analysis of the different communication style scores as well as the results obtained in the attention tests (Table 1). Regarding communication styles, we highlight how the assertive communication style predominated in our study (M = 16.63; SD = 6.06). Levene’s test was carried out in order to observe if the variances of both groups were equal. The data presented significant differences between the variances; therefore, the use of non-parametric tests was recommended in the data analysis. First, we carried out the Mann–Whitney U statistic (Table 2), dividing the participants according to sex. We found differences in the assertive style (Z = −2.04, *p* < 0.05), the passive style (Z = −3.21, *p* < 0.05) and sex, with girls showing higher scores in assertive communication style than boys; girls also showed less frequent passive communication styles.

Next, we carried out the same procedure, but this time dividing the participants according to grade (Table 3). Here, we found significant differences between the groups in both the assertive communication style (z = −4.73, *p* < 0.05) and the aggressive style (Z = −3.11, *p* < 0.05), but there were no significant differences between groups with regard to passive style. In order to determine the existence of relationships between communication styles and attention, we calculated the Spearman’s correlation coefficient (Table 4) with communication style score and the results obtained in the SDMT and the d2 attention test.

A statistically significant and proportional correlation is found between the assertive style and the total effectiveness in the test (r_s_ = 0.230, *p* < 0.05). A positive and statistically significant correlation is also observed between assertiveness and the number of correct answers on the SDMT (r_s_ = 0.234, *p* < 0.01). The aggressive communication style shows a negative and statistically significant correlation with the correct answers on the SDMT (r_s_ = −0.227, *p* < 0.05). Similarly, the aggressive style also positively correlates with the number of errors (r_s_ = 0.246, *p* < 0.05).

We also wanted to analyze the existence of differences in attention tests with regard to sex, not finding significant differences, as shown in Table 5.

When we carried out the same analysis, comparing the different grade groups (third and sixth grades), we did find significant differences for some of the indices. We could see how the sixth grade group had more correct answers than the third grade group in the SDMT (z = −4.02, *p* < 0.05). Regarding the d2 test, sixth graders gave more answers than third graders (Z = −4.39, *p* < 0.05), they made fewer commissions (z = −2.28, *p* < 0.05) and more omissions (z = −2.34, *p* < 0.05), and showed better effectiveness in the test (Z = −4.54, *p* < 0.05) and better concentration (Z = −2.46, *p* < 0.05) (Table 6).

## 4. Discussion

Although various authors, such as Rebollo and Montil [60] or Barkley [61], were able to postulate that attentional alterations could be a symptom of impaired executive functioning, in our work, we focus on attention that is considered an independent and differentiated executive function.

In the present study, we analyzed whether assertive behavior or different communication styles may be related to attention span. At first, we observed how, in general, girls showed greater assertiveness than boys. The passive communication style was less frequent in girls than in boys, while the aggressive style showed no differences.

We can see how the assertive style is more frequent in older children and how the aggressive style of communication is also less frequent in this group. On the other hand, passive behavior did not show significant changes between the two age groups, showing fewer changes.

Attention also seemed to also improve over time with results that are in line with those obtained by Jiménez et al. [62], who observed better performance as a function of age.

In our study, we observed how those children who had better attention scores also had better assertiveness results. Specifically, children who scored higher on the assertiveness scale had better results in SDMT and they were able to work in a faster way, offering better overall effectiveness on the test than those who showed passive or aggressive styles.

Authors such as Pérez and Lago [35,36] or García [37], who refer to the fact that, in assertive behavior, attention is a behavioral process that could facilitate interaction with our environment and allow for receiving adequate information from those around us, we can understand why younger children show less assertive behavior. In fact, we can mention how seven-year-olds are slower when they have to redirect attention from one ear to another in dichotic listening tasks, while from the age of 11, most children are able to shift their focus from one message to another almost as quickly as adults [63]. Therefore, the improvement in attention would also imply an improvement in assertiveness, as our study showed.

In the two smaller groups of children, we observed how, although we did not obtain significant differences, they presented more commissions (number of marked irrelevant elements) than the older ones as an indication that the younger students were affected by the presence of distracting stimuli. This aspect changes with age, as Jimenez et al. [62] also showed. This fact would also explain the poorer results in assertiveness in younger children because they cannot cope well with stimuli from the environment.

These results are in line with a study by Wheeler Maedgen and Carlson [39] on ADHD children, where a relationship between inattention and lack of assertive behavior was observed. It is striking that, although there were no differences in attention performance according to sex, there were differences in assertive behavior, which is why we infer that attention does affect communication styles and that we should continue in this line of work to try to describe what other aspects influence assertive communication skills in a significant way, as attention does. Therefore, we observed how assertive children were receptive to the stimuli of their environment, while passive children preferred to stay out of the way and reject new incentives. The most aggressive ones, however, opted to control the situation and did not focus on a single stimulus, but rather tried to cover as much as possible, and hence, they could not respond correctly to everyone.

It would be interesting to assess why this maintenance of passive behaviors occurs over time, which is perhaps less related to attentional aspects. In the same way that improved attention implies a decrease in aggressiveness, it would also be interesting to observe what other processes are related to passive behavior and communication style.

The time stability of the passive style suggests that, although certain aspects related to education or maturation itself may influence the development of assertive and aggressive behaviors, they do not seem to have an effect on passive style children, which opens the door to study what other factors could influence this communication style in order to be able to create programs for their improvement and possible later implications and complications.

Like Albert et al. [63], we consider that when it comes to intervening in attention problems, programs usually focus on cognitive aspects, paying less attention to intervention in other key aspects of development, such as social relations. This is why we think that the development of research that sheds more light on the possible relationships between communication styles and attention should be pursued since they could offer benefit by improving children’s social skills and attention. Therefore, if the attention deficit is significantly related to social skills [64], it would be possible to think that an improvement in these skills could also influence an improvement in attention. It would be interesting to address the development of communication styles in the first school cycles since differences could be observed at later ages. Although it is true that family traits can influence children’s communication styles, this was not the central part of our study, which is based on knowing the relationships that communication styles have with aspects of cognitive processing (attention), but it can be detected as a limitation in the current study.

## 5. Concluding Remarks

Children with an assertive communicative style had a greater attention span than children with a passive or aggressive communication style; this assertiveness increased with age. Girls showed more assertive behaviors than boys as well as fewer passive behaviors and communication strategies within the age range of our study. The communication style had an effect on all the students in the third and sixth grades. Gender did not have an effect on attention capacity. It would be interesting in the future to carry out studies with wider age ranges and including longitudinal studies to check the evolution of these styles over the years.

## Figures and Tables

**Table 1:** Descriptive analysis of communication styles (assertive, passive and aggressive) and attention indices in our study population.

Communication Style Attention Tests	Mean	Median	Mode	SD	*n*
Assertive	16.63	19.00	22.00	6.06	77
Passive	3.20	3.00	1.00	2.59	77
Aggressive	1.39	0.00	0.00	2.47	77
HIT SDMT	38.68	39.00	35.00	12.37	77
SDMT-ERRORS	1.01	0.00	0.00	2.48	77
D2-TR	262.48	251.00	211.00	103.95	77
D2-TA	80.90	78.00	66.00	31	77
D2-C	11.63	3.00	1.00	21.05	77
D2-OR	30.46	13.00	2.00	44.17	77
D2-TOT	219.01	210.00	206.00	76.01	77
D2-CON	66.58	68.00	62.00	43.66	77

Note: HIT SDMT, correct score for SDMT; SDMT Errors, errors in SDMT; D2-TR, total responses; D2-TA, total hits, number of relevant elements; D2-C, commissions, number of irrelevant items marked; D2-OR, omissions, number of relevant elements attempted but not marked; D2-TOT, total effectiveness in the test, that is, TR − (C + OR); D2-CON, concentration index, TA-C.

**Table 2:** Mann–Whitney U test results for sex.

Communication Style	Sex (*n*)	Mean Rank	z	sig
Assertive	Girls (40)	17.72	−2.04	0.041
	Boys (37)	15.45		
Passive	Girls (40)	2.27	−3.21	<0.001
	Boys (37)	4.21		
Aggressive	Girls (40)	0.92	−1.43	0.153
	Boys (37)	1.89		

**Table 3:** Mann-Whitney U test results for class groups.

Communication Style	Course (*n*)	Mean Rank	z	sig
Assertive	3° (40) 6° (37)	27.44 51.5	−4.73	<0.001
Passive	3° (40) 6° (37)	38.86 39.15	−0.05	0.95
Aggressive	3° (40) 6° (37)	45.8 31.65	−3.11	0.002

**Table 4:** Spearman’s correlation coefficient between communication styles and attention indices.

	Assertive	Passive	Aggressive	HIT SDMT	MISTAKES SDMT	D2-TR	D2-TA	D2-C	D2-OR	D2-TOT
Assertive	1									
Passive	−0.393 **	1								
Aggressive	−0.528 **	−0.018	1							
HIT SDMT	0.234 *	0.015	−0.227 *	1						
MISTAKES SDMT	−0.074	0.029	0.246 *	−0.225 *	1					
D2-TR	0.192	0.222	−0.171	0.353 **	−0.139	1				
D2-TA	0.067	0.043	0.114	0.393 **	−0.087	0.412 **	1			
D2-C	−0.197	0.191	0.197	−0.252 *	0.043	−0.009	−0.208	1		
D2-OR	0.1	0.089	−0.123	−0.06	0.109	0.453 **	−0.369 **	0.247 *	1	
D2-TOT	0.230 *	0.114	−0.131	0.445 **	−0.134	0.887 **	0.605 **	−0.250 *	0.231 *	1
D2-CON	0.182	−0.029	−0.026	0.492 **	−0.157	0.306 **	0.844 **	−0.449 **	−0.460 **	0.583 **

Notes: HIT SDMT, correct score in SDMT; Errors SDMT, errors in SDMT; TR, total responses; TA, total hits, number of relevant elements; C, commissions, number of irrelevant items marked; OR, omissions, number of relevant elements attempted but not marked; TOT, total effectiveness in the test, that is, TR − (C + OR); CON, concentration index, TA-C. ** The correlation is significant at the 0.01 level (2 tails). * The correlation is significant at the 0.05 level (2 tails).

**Table 5:** Mann-Whitney U test results for attention and sex. Hit SDMT, correct score in the SDMT; Mistakes SDMT, errors in the SDMT; TR, total responses; TA, total hits, number of relevant elements; C, commissions, number of irrelevant items marked; OR, omissions, number of relevant elements attempted but not marked; TOT, total effectiveness in the test, that is, TR − (O + C); CON, concentration index, TA-C.

Attention Tests	Sex (*n*)	Mean Rank	z	sig
HIT SDMT	Girls (40)	40.18	−0.48	0.63
	Boys (37)	37.73		
MISTAKES SDMT	Girls (40)	36.28	−1.32	0.18
	Boys (37)	41.95		
D2-TR	Girls (40)	39.14	−0.05	0.95
	Boys (37)	38.85		
D2-TA	Girls (40)	34.35	−1.89	0.05
	Boys (37)	44.03		
D2-C	Girls (40)	37.76	−0.50	0.61
	Boys (37)	40.34		
D2-OR	Girls (40)	42.75	−1.53	0.12
	Boys (37)	34.95		
D2-TOT	Girls (40)	38.59	−0.16	0.86
	Boys (37)	39.45		
D2-CON	Girls (40)	36.80	−0.89	0.37
	Boys (37)	41.38		

**Table 6:** Mann-Whitney U test results for attention and class groups (3° & 6°).

Attention Tests	Course (*n*)	Mean Rank	z	sig
HIT SDMT	3 (40) 6 (37)	29.15 49.65	−4.02	**<0.001**
MISTAKES SDMT	3 (40) 6 (37)	39.25 38.73	−0.12	0.903
D2-TR	3 (40) 6 (37)	28.24 50.64	−4.39	**<0.001**
D2-TA	3 (40) 6 (37)	36.98 41.19	−8.26	0.409
D2-C	3 (40) 6 (37)	44.56 32.99	−2.28	**0.022**
D2-OR	3 (40) 6 (37)	33.26 45.2	−2.34	**0.019**
D2-TOT	3 (40) 6 (37)	27.85 51.05	−4.54	**<0.001**
D2-CON	3 (40) 6 (37)	32.96 45.53	−2.46	**0.014**

Note: Hit SDMT, correct score in the SDMT; Mistakes SDMT, errors in the SDMT; TR, total responses; TA, total hits, number of relevant elements; C, commissions, number of irrelevant items marked; OR, omissions, number of relevant elements attempted but not marked; TOT, total effectiveness in the test, that is, TR − (O + C); CON, concentration index, TA-C.

## Data Availability

Data will be available upon request.
